# Synthetic microbial community to enhance the fermentation and feed valorization of pineapple waste

**DOI:** 10.3389/fmicb.2026.1929714

**Published:** 2026-09-24

**Authors:** Kuanmin Yang, Ju Qin, Xiaoqiang Xue, Guozhao Lin, Kaiyong Huang, Dongdi Gu, Jiliang Su, Jun Wang, Yiyan Cui, Xianyong Ma, Zhimei Tian, Miao Yu, Zhichang Liu, Ting Rong, Yusheng Lu, Ruqing Zhong, Xiangdong Liu, Pan Yang, Dun Deng

**Affiliations:** 1 Shenzhen Kingkey Smart Agriculture Times Co., Ltd., Shenzhen, China; 2 Institute of Animal Science, Guangdong Academy of Agricultural Sciences, State Key Laboratory of Swine and Poultry Breeding Industry, Key Laboratory of Animal Nutrition and Feed Science in South China, Ministry of Agriculture, Guangdong Public Laboratory of Animal Breeding and Nutrition, Guangdong Engineering Technology Research Center of Animal Meat Quality and Safety Control and Evaluation, Guangzhou, China; 3 Institute of Agricultural Resources and Environment, Guangdong Academy of Agricultural Sciences, Guangzhou, China; 4 Institute of Animal Science, Chinese Academy of Agricultural Sciences, Beijing, China; 5 Guangxi Yangxiang Co., Ltd., Guangxi, China

**Keywords:** anti-nutritional factors degradation, fermentation, fermented pineapple waste, pigs, synthetic microbial community

## Abstract

Global livestock industry is confronted with severe feed-supply pressure, and valorizing agro-industrial by-products has become a promising way to alleviate feed resource shortages. Large quantities of pineapple waste are produced annually during pineapple processing, but antinutritional factors greatly limit its direct application in pig diets. Pineapple waste (PW) is a promising agro-industrial by-product for feed production; however, its high levels of antinutritional factors and structural carbohydrates limit its utilization in monogastric animals. This study aimed to develop a synthetic microbial community (SysComs) for upgrading PW into a functional feed ingredient. Four functional strains were separately screened according to their dominant single degrading or metabolite-synthesizing traits, and further combined into a synthetic community owing to their complementary metabolic capacities, including tannin-degrading *Lactiplantibacillus plantarum*, xylan-degrading *Pediococcus pentosaceus*, phytase-producing *Lactobacillus fermentum*, and butyrate-producing *Clostridium butyricum*. Fermentation with SysComs markedly improved the nutritional and functional properties of PW. Compared with raw PW, fermented pineapple waste (FPW) showed substantial reductions in tannin and phytate contents, with removal rates reaching 80.65 and 89.62%, respectively. Total dietary fiber and insoluble dietary fiber were significantly decreased, whereas soluble dietary fiber increased from 5.60 to 8.18%. Meanwhile, butyrate concentration increased from 1.65 to 6.63%, accompanied by a decrease in pH from 6.10 to 4.87. Microbial community analysis showed that SysComs fermentation shifted PW from a contaminant-rich microbial community toward a lactic acid bacteria-dominated structure, with enrichment of *Lactobacillus*, *Levilactobacillus*, *Enterococcus*, *Bacillus*, and *Weissella*. Untargeted metabolomics further revealed that fermentation extensively remodeled the metabolite profile of PW, with 258 metabolites upregulated and 437 downregulated. Pathways related to flavonoid biosynthesis, flavone and flavonol biosynthesis, and secondary metabolite biosynthesis were significantly enriched. Luteolin, taxifolin, phlorizin, and gallocatechin were markedly increased. In pig experiment, dietary FPW supplementation significantly increased average daily gain by 13.10% and improved gross energy digestibility from 85.36 to 87.46% compared with the control group (*p* < 0.05). Moreover, FPW reshaped the colonic microbiota by increasing microbial richness and enriching beneficial fiber-degrading and short-chain fatty acid-associated taxa, including *Christensenellaceae* R-7 group, *Lachnospiraceae* NK4A136 group, and *Monoglobus*. These findings demonstrate that SysComs fermentation effectively reduced antinutritional factors, enhanced nutrient accessibility, enriched functional metabolites, and improved growth performance and gut microbial ecology in pigs. Targeted microbial fermentation is an effective strategy for converting pineapple waste into a high-value functional feed ingredient.

## 1 Introduction

Food security remains a global priority, particularly as the demand for feed resources continues to rise alongside the rapid development of the livestock industry (Makkar, 2018; Food and Agriculture Organization of the United Nations, 2023). Feed grains constitute a substantial proportion of total food consumption, and the increasing competition between human food and animal feed has intensified pressure on conventional feed resources (Makkar, 2018). In this context, reducing reliance on traditional feed grains such as corn and soybean, while developing alternative, sustainable feed resources, has become essential for ensuring long-term food security and promoting the sustainable development of animal production systems. Agricultural byproducts, characterized by their low cost, wide availability, and non-competition with human food, represent promising alternatives for feed applications (Makkar, 2018).

Pineapple processing generates large quantities of pineapple waste (PW), which is often underutilized or improperly disposed, leading to environmental concerns (Sukri et al., 2023). PW has considerable potential as a feed resource for rich carbohydrates and nutrients. However, its practical application is limited by the presence of anti-nutritional factors, including tannins, phytic acid, and high levels of structural fiber (Sukri et al., 2023; Kiatti et al., 2023). Anti-nutrients reduce the digestibility of nutrients, suppress animal growth performance, and hinder the efficient utilization of PW in animal diets. For instance, tannins can bind proteins and digestive enzymes, Hexaphosphoric acid can be combined with essential minerals, and excessive fiber limits nutrient absorption (Gundersen et al., 2022; Okaiyeto et al., 2025). Therefore, effective strategies to reduce these anti-nutritional factors are crucial for improving the feed value of PW. Microorganisms play a central role in the bioconversion of agricultural by-products, serving as essential biological agents for substrate degradation and value enhancement (Emkani et al., 2022; Sooraj et al., 2025). Due to their diverse enzymatic systems and metabolic versatility, microorganisms have been widely applied to improve the nutritional quality of low-value agricultural residues and lignocellulosic biomass (Bai et al., 2020). One of the major limitations in utilizing agricultural by-products as feed resources is the presence of anti-nutritional factors, such as tannins, phytic acid, and complex fiber fractions, which reduce nutrient digestibility, impair feed utilization, and restrict nutrient availability (Samtiya et al., 2020). Microbial fermentation has emerged as an effective strategy to address these constraints through the secretion of functional enzymes, including tannase, phytase, cellulase, and xylanase, which degrade anti-nutritional compounds and break down structural carbohydrates, thereby improving substrate digestibility and nutrient accessibility (Xie et al., 2024; Yadav et al., 2025). Beyond substrate degradation, microbial fermentation also generates beneficial metabolites that further enhance the functional value of agricultural by-products, among which butyrate has attracted particular attention. As an important short-chain fatty acid, butyrate is recognized for its positive effects on intestinal health, epithelial integrity, anti-inflammatory regulation, and energy metabolism in animals (Ma et al., 2024). Certain microorganisms can directly produce butyrate during fermentation or promote metabolic pathways favorable for butyrate accumulation, thereby increasing the biological value of fermented feed ingredients. Compared with other organic acids, butyrate provides not only preservative effects through acidification and inhibition of undesirable microorganisms, but also serves as a functional metabolite with direct physiological benefits to the host. In addition, different microbial species exhibit complementary metabolic characteristics; for example, *Bacillus* species are highly efficient in secreting extracellular enzymes for macromolecule degradation, while selected fermentative bacteria contribute to beneficial metabolite production, including butyrate synthesis (Yang, S., et al., 2025; Yang, X., et al., 2025). Therefore, the strategic use of functional microorganisms or microbial consortia represents a promising and sustainable approach for upgrading agricultural by-products into nutritionally enhanced and biologically active feed resources, contributing to waste valorization and sustainable livestock production.

Despite the potential value of fermented pineapple waste as an alternative feed resource, few studies have systematically constructed synthetic microbial communities to upgrade pineapple waste, and comprehensive evidence regarding its nutritional transformation, metabolite remodeling, and animal-feeding effects remains insufficient. Therefore, developing an efficient bioconversion strategy for PW is of both scientific and practical significance.

Therefore, this study aimed to screen strains with target functional traits to assemble a synthetic microbial community (SysComs). This consortium was then applied for the fermentation of pineapple waste (PW), to characterize shifts in its nutritional components, antinutritional factors, and metabolite profiles triggered by SysComs-driven fermentation. Furthermore, we evaluated the effects of fermented pineapple waste (FPW) on growth performance, nutrient digestibility, and colonic microbiota in growing pigs. This work provides theoretical support for the high-value utilization of pineapple waste and promotes the development of circular feeding systems.

## 2 Materials and methods

### 2.1 Ethics statement

This study was approved by the Animal Care and Use Committee of the Institute of Animal Science, Guangdong Province Academy of Agricultural Sciences, under approval number GQ2025018.

### 2.2 Screening and functional strains

In this study, all bacterial strains used were obtained from the laboratory’s microbial strain collection. A 2% inoculum was applied to the corresponding screening medium for subsequent experiments. The medium for screening tannins-degrading strains (per liter) was modified from the basal medium to include (NH_4_)_2_SO_4_ 2.0 g, NaCl 0.3 g, FeSO_4_·7H_2_O 0.03 g, K_2_HPO_4_ 1.0 g, MgSO_4_·7H_2_O 0.03 g, CaCl_2_ 7.5 g, supplemented with 10.0 g tannins as the substrate and 1.0 g glucose to support growth. For screening xylan-degrading bacteria, the medium included (per liter): yeast extract 3.0 g, NaCl 0.3 g, K_2_HPO_4_ 1.0 g, MgSO_4_·7H_2_O 1.0 g, CaCl_2_ 7.5 g, with 5.0 g xylan as the sole carbon source. The medium for screening phytate-degrading bacteria consisted of (per liter): (NH_4_)_2_SO_4_ 2.0 g, NaCl 0.3 g, K_2_HPO_4_ 1.0 g, MgSO_4_·7H_2_O 0.03 g, CaCl_2_ 7.5 g, supplemented with 3.0 g sodium phytate and 5.0 g glucose. For screening high butyrate-producing bacteria, the medium contained (per liter): glucose 20.0 g, corn steep liquor 3.7 g, (NH_4_)_2_SO_4_ 1.0 g, NaHCO_3_ 1.24 g, MnSO_4_·H_2_O 0.2 g, MgSO_4_·7H2O 0.2 g, and CaCl_2_ 0.02 g under anaerobic conditions. The effectiveness of tannins degradation by bacteria was evaluated by measuring gallic acid production after incubation. In addition, xylan (Aladdin, Shanghai) degradation by bacteria was assessed by determining reducing sugar release using the dinitrosalicylic acid method. The international unit (U) of enzyme activity is defined as the amount of enzyme required to produce 1 μmol of reducing sugar per minute. Phytate degradation by bacteria was evaluated based on phytase activity, which was assayed with the ferrous sulfate-molybdenum blue method. For butyrate production by bacteria, the concentration of butyric acid in the fermentation broth was quantified after 48 h. The strains exhibiting the highest substrate degradation rate or butyrate production were selected for further experiments. The concentration of 7.5 g/L calcium chloride was specially formulated to counteract the chelating inhibitory effects of tannins and phytic acid. Meanwhile, this consistent dosage unifies the culture conditions for parallel screening, stabilizes the spatial structure of extracellular degrading enzymes, and maximizes the detectable activity of degrading enzymes produced by strains.

### 2.3 Experimental materials and fermentation method

PW was obtained from the Zhanjiang Kingkey Smart Agriculture Times Co., Ltd. (Zhanjiang, China). The PW used as raw material was sourced from locally harvested pineapples in Guangdong Xuwen in August. Fresh PW were collected after processing. The raw PW were first pressed to maintain an initial moisture content of 55–60%. After fermentation, the moisture content of FPW was 78.49%. Then the PW/FPW samples were evenly sprayed with a mixed solution of a synthetic microbial community, and packed into double-layer fermentation bags. Each fermentation batch weighed approximately 1,000 kg and was sealed and fermented at an average temperature of 47.3 °C for 3 days. The nutritional composition of the PW before and after fermentation is shown in Table 1.

**Table 1:** Nutritional value of fermented pineapple waste (as-fed basis).

Items	Unfermented	Fermented	*p* value
CP, %	9.15 ± 0.08	9.09 ± 0.04	0.56
EE, %	0.62 ± 0.02	0.61 ± 0.04	0.37
Total dietary fiber, %	43.64 ± 0.19	41.57 ± 0.54	0.03
Insoluble dietary fiber, %	38.04 ± 0.61	33.39 ± 0.32	0.02
Soluble dietary fiber, %	5.60 ± 0.56	8.18 ± 0.47	0.01
Ash, %	4.64 ± 0.11	4.85 ± 0.17	0.40
Butyrate, %	1.65 ± 0.19	6.63 ± 0.16	<0.01
Phytates, mg/100 g	1.83 ± 0.10	0.19 ± 0.05	<0.01
Tannins, mg/100 g	12.56 ± 1.38	2.43 ± 0.65	<0.01
pH	6.1 ± 0.24	4.87 ± 0.38	0.02

### 2.4 Determination of tannins, phytates, pH, and chemical composition

The tannin content was determined using the Folin–Ciocalteu colorimetric method with a microplate reader (Spectra Max M5; Molecular Devices LLC, San Jose, CA, USA). The phytate content was analyzed following the colorimetric method based on ferric ion precipitation as described by standard procedures. The pH value was measured using a calibrated digital pH meter. The contents of crude protein, ether extract, and ash were determined according to the standard methods described by Cui et al. (2025). The contents of total dietary fiber, insoluble dietary fiber, and soluble dietary fiber were determined according to the standard methods (AOAC 2011.25) (McCleary et al., 2012).

### 2.5 Microbial diversity analysis

Microbial community diversity was analyzed using high-throughput sequencing of the 16S rRNA gene. Total genomic DNA was extracted from samples using a commercial DNA extraction kit according to the manufacturer’s instructions. The V3-V4 hypervariable region of bacterial 16S rRNA gene was PCR performed using universal PCR method. The PCR amplification and subsequent bioinformatic analyses were performed by Shanghai Majorbio Bio-Pharm Technology Co., Ltd. Sequencing was conducted on an Illumina platform, and the raw reads were quality-filtered, merged, and processed to obtain high-quality clean tags. Operational taxonomic units (OTUs) are clustered with a sequence similarity of 97%, and taxonomic assignment was performed against a reference database. Alpha diversity indices and beta diversity analyses were calculated to evaluate microbial richness and community structure differences among samples. The data were analysed using the Majorbio Cloud Platform.^1^

### 2.6 Metabolomics assay

Using the Vanquish UHPLC system combined with the Q Exactive HF-X mass spectrometer (Thermo Fisher Scientific, Waltham, Massachusetts, USA) for metabolic analysis of the sample, equipped with a Hypersil GOLD column (Thermo Fisher Scientific). All samples were subjected to standardized procedures for preprocessing, extraction, identification, and analysis according to previously described methods (Yuan et al., 2012). For each metabolite, the differences between groups were evaluated using fold change and the variable importance in projection (VIP) score derived from the orthogonal partial least squares discriminant analysis (OPLS-DA) model. The VIP value represents the contribution of each variable to the model, with higher values indicating greater importance. Metabolites with VIP ≥ 1.5, *p* < 0.05, and |log2fold change| greater than 1.5 were defined as significantly differential metabolites.

### 2.7 Animal experimental design, diets and management

A total of 552 pigs aged 73 days were randomly assigned to three groups, each with four replicates of 46 pigs per replicate. The control group was fed a basic diet. The PW group was fed with basic feed +3.5% unfermented pineapple waste. The FPW group was fed a basic diet of +3.5% fermented pineapple waste. The experimental diets were formulated according to the nutrient requirements recommended by Nutrient Requirements of Swine (GB/T 39235-2020; National Standardization Management Committee, 2026a). The composition of the experimental diets is shown in Table 2. Pigs had ad libitum access to feed and water and were managed according to standard operating procedures.

**Table 2:** The composition of experimental diets (as-fed basis, %).

Ingredient	CON	PW	FPW
Stage-1			
Corn	30.00	30.00	30.00
Rice bran	25.00	25.00	25.00
Sorghum	15.10	14.18	14.18
43% soybean meal	13.42	13.57	13.57
Wheat flour	6.00	6.00	6.00
Fermented soybean meal	6.00	3.00	3.00
Premix	1.25	1.25	1.25
Dicalcium phosphate	0.88	0.88	0.88
Limestone	0.87	0.87	0.87
Sodium chloride	0.40	0.40	0.40
L-lysine sulfate (70%)	0.49	0.49	0.49
Dl-methionine	0.37	0.37	0.37
Soybean oil	0.16	0.43	0.43
Choline chloride (75%)	0.07	0.07	0.07
PW		3.50	
FPW			3.50
Total	100	100	100
Calculated content (%)			
Crude protein (CP), %	16.5	16.5	16.4
Net energy, kcal/kg	2,509	2,509	2,497
SID-Lys, %	0.9	0.9	0.9
SID-Met, %	0.3	0.3	0.3
SID-Thr, %	0.7	0.7	0.6
SID-Trp, %	0.2	0.2	0.2
Stage-2			
Corn	40.00	40.00	40.00
Barley	20.36	16.77	16.77
43% Soybean meal	11.73	12.41	12.41
Rice bran	10.00	10.00	10.00
Wheat flour	7.46	9.79	9.79
Fermented soybean meal	5.00	2.00	2.00
Premix	1.25	1.25	1.25
Soybean oil	0.95	1.05	1.05
Dicalcium phosphate	0.83	0.89	0.89
Limestone	0.83	0.79	0.79
Sodium chloride	0.40	0.40	0.40
L-lysine sulfate (70%)	0.54	0.52	0.52
DL-methionine	0.38	0.38	0.38
Threonine	0.21	0.20	0.20
Choline chloride (75%)	0.07	0.07	0.07
PW		3.50	
FPW			3.50
Total	100.00	100.00	100.00
Calculated content (%)			
Crude protein (CP), %	15.5	15.5	15.2
Net energy, kcal/kg	2,500	2,500	2,485
SID-Lys, %	0.9	0.9	0.9
SID-Met, %	0.3	0.3	0.3
SID-Thr, %	0.6	0.6	0.6
SID-Trp, %	0.2	0.2	0.2

### 2.8 Growth performance and sample collection

During the experiment, pigs were weighed at both the beginning and the end of the trial following a 12 h fasting period, and the initial and final body weights were recorded. Feed intake was measured daily on a per-replicate basis. Growth performance was assessed by calculating average daily gain (ADG), average daily feed intake (ADFI), and feed conversion ratio (FCR). Diet samples were collected for each group in accordance with the GB/T 14699-2023 (National Standardization Management Committee, 2026b), using the quartering method. Feed and fecal samples were collected from each pen, placed in sealed bags, and stored at −20 °C until further analysis.

### 2.9 Data processing and statistical analysis

All data are expressed as mean ± standard error of the mean (SEM). Statistical analyses were performed using JMP software. The Shapiro–Wilk test implemented in JMP software was used to assess normality, and all datasets satisfied the assumptions required for parametric analysis (*p* > 0.05). Evaluate inter group differences through one-way analysis of variance (ANOVA), and then conduct *post hoc* analysis through Tukey’s multiple comparison test. Statistical significance was set at *p* < 0.05, and 0.05 ≤ *p* < 0.10 was considered a tendency. Graphical visualization, including bar charts and line graphs, was conducted using GraphPad Prism.

## 3 Results and discussion

### 3.1 Screening and characterization of functional strains

To construct a synthetic microbial community for pineapple waste valorization, candidate strains were first screened via single-function indicators (tannin degradation, xylanase activity, phytase production, butyrate synthesis). The four strains with optimal respective functions were then selected and mixed as a consortium because their metabolic pathways could complement each other to remove multiple anti-nutritional factors simultaneously (Figure 1). The degradation of tannins—a primary anti-nutritional factor in fruit residues—varied significantly among the tested isolates (Figures 1A,B). Compared to the control group (CK), strain T3 (*Lactiplantibacillus plantarum*) demonstrated the most robust degradation capacity, reducing the tannoid concentration from 1,500 mg/L to 295.4 mg/L. This represents a peak degradation rate of approximately 80.31% (Figure 1B), significantly higher than other candidates (*p* < 0.05). Consequently, T3 was identified as a critical functional isolate for mitigating the inhibitory effects of tannins during the fermentation process.

**Figure 1:**
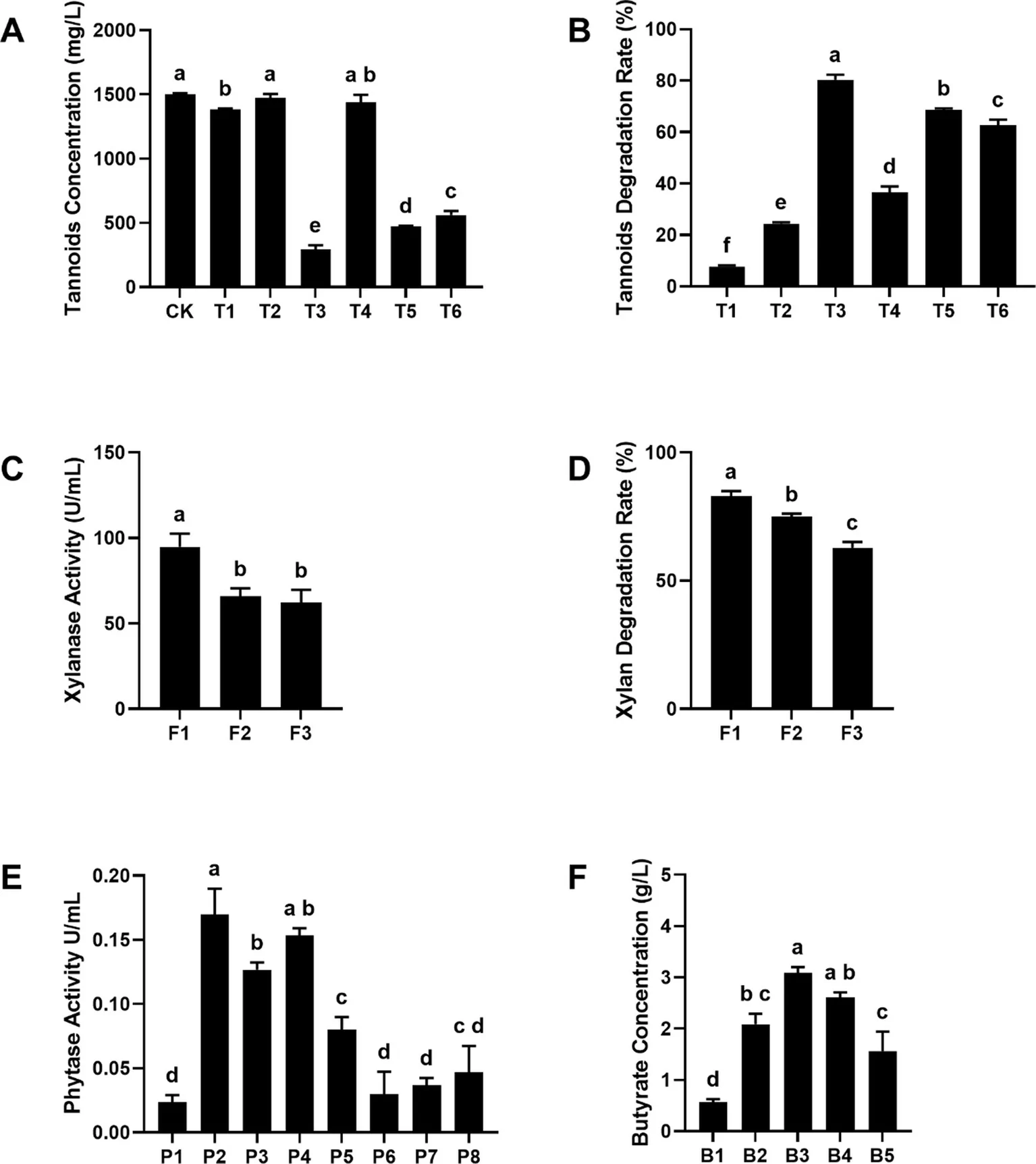
Screening and functional characterization of strains for pineapple waste valorization. **(A)** Determination of tannins degradation ability of candidate strains; **(B)** Tannins degradation rate of different treatments; **(C)** Determination of xylanase activity of selected strains; **(D)** Xylan degradation rate of functional microbial groups; **(E)** Determination of phytase activity of screened strains; **(F)** Butyrate concentration produced by fermentation strains.

The fiber-degrading potential of the isolates, essential for improving feed digestibility, was evaluated through xylanase activity and xylan degradation efficiency. Figure 1C, strain F1 (*Lactobacillus fermentum*) exhibited superior xylanase secretion, achieving an activity of 94.80 U/mL, significantly outperforming F2 and F3 (*p* < 0.05). This enzymatic efficiency translated into a high xylan degradation rate of 82.93% (Figure 1D). Furthermore, the phosphorus-releasing capability was assessed via phytase activity; Strain P2 (*Pediococcus pentosaceus*) showed the highest yield at 0.17 U/mL, suggesting its potential to enhance mineral bioavailability in the final feed product (Figure 1E).

Finally, the probiotic potential of the candidates was assessed by quantifying butyrate production (Figure 1F). Strain B3 (*Clostridium butyricum*) emerged as the most efficient producer, Generate a concentration of 3.08 g/L butyrate. Based on these multidimensional screening results—specifically the high tannoid degradation of T3, the robust xylanolytic activity of F1, the superior phytase production of P2, and the butyrate-yielding capacity of B3—these four strains were strategically selected as the core components for the formulation of the synthetic microbial consortia.

### 3.2 Fermentation of pineapple waste by SysComs

The preparation of the fermented feed is shown in Figure 2. The nutritional profile of pineapple waste underwent significant transformation following fermentation by the synthetic microbial community (Table 1). While the levels of crude protein (CP) and ether extract (EE) are no significant change, the structural carbohydrates and anti-nutritional factors were substantially modified. Specifically, total dietary fiber (TDF) decreased from 43.64 to 41.57% (*p* = 0.03), primarily driven by a significant reduction in insoluble dietary fiber (IDF), which dropped from 38.04 to 33.39% (*p* = 0.02). Conversely, Soluble dietary fiber (SDF) content ranges from 5.60 to 8.18% (*p* = 0.01). This shift from insoluble to soluble fractions suggests that the SysComs effectively degraded complex cell wall polysaccharides into more fermentable forms. Furthermore, fermentation led to a dramatic reduction in anti-nutritional factors, with phytate levels plummeting from 1.83 mg/100 g to 0.19 mg/100 g and tannin content decreasing from 12.56 mg/100 g to 2.43 mg/100 g (*p* < 0.01). A concomitant rise in butyrate concentration (from 1.65 to 6.63%, *p* < 0.01) and a significant decline in pH (from 6.1 to 4.87, *p* = 0.02) were also observed. Given the effective reduction of tannins and phytates alongside the enrichment of butyrate and SDF, this fermented pineapple waste was selected for subsequent valorization trials.

**Figure 2:**
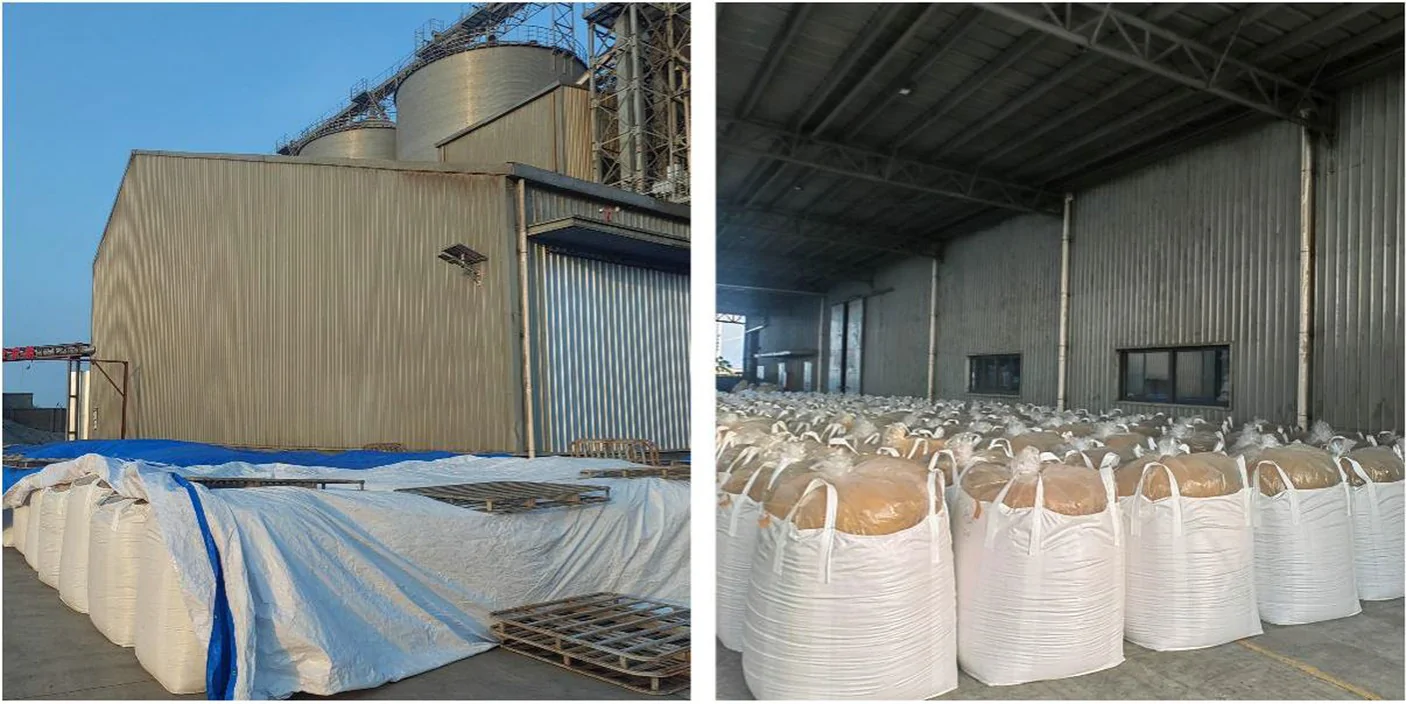
Fermented feed production using SysComs.

The observed decrease in pH was largely attributed to the robust metabolic activity of the synthetic community, which utilized the carbohydrate matrix to synthesize organic acids (Atasoy et al., 2024). This acidic environment is characteristic of successful fermentation and helps in inhibiting pathogenic growth while enhancing storage stability (Atasoy et al., 2024). The conversion of IDF to SDF and the overall reduction in TDF reflect the potent cellulolytic and xylanolytic activities of the inoculated strains, likely similar to the action of *Bacillus* species known for secreting fiber-degrading enzymes (Khongkool et al., 2025). The significant increase in butyrate levels further underscores the metabolic efficiency of the selected functional strains, such as the butyrate-producing isolates identified in the screening phase (Zhang et al., 2024; Clark et al., 2021).

Consistent with our findings, previous studies have demonstrated that microbial fermentation can significantly mitigate anti-nutritional factors like tannins and phytates in agricultural by-products (Khongkool et al., 2025; Joudaki et al., 2023). The reduction in tannins may be attributed to the secretion of tannase or other extracellular enzymes by the SysComs that break down complex polyphenols into simpler, less inhibitory compounds (Chávez-González et al., 2018). Similarly, the sharp decline in phytates is likely due to high phytase activity within the consortia, which facilitates the hydrolysis of phytic acid and improves mineral bioavailability (Joudaki et al., 2023). The enzymatic degradation of these macromolecules not only detoxifies the substrate but also releases nutrients that support microbial proliferation and the accumulation of beneficial metabolites like SCFAs (Zhang et al., 2024; Khongkool et al., 2025).

### 3.3 Bacterial diversity and community composition

The number of sequences obtained for individual treatments was 40,651. An overall total of 130 operational taxonomic units (OTUs) were clustered at the 97% similarity level for further analysis (Xu et al., 2024; Li et al., 2022). Alpha diversity analysis revealed that the Observed species, ACE, and Chao1 indices significantly decreased in the FPW compared with the PW, indicating a reduction in overall microbial richness following the fermentation process. Conversely, increases in the Simpson and Shannon indices were observed in FPW, reflecting a more balanced and even distribution among the dominant microbial populations (Zhang et al., 2023; Yang et al., 2025; Yang et al., 2025). At the phylum level, while *Pseudomonadota*, *Bacteroidota*, *Bacillota*, and *Cyanobacteriota* remained the primary taxa, the relative abundance of *Bacillota* increased markedly to become dominant in FPW, displacing the *Cyanobacteriota* that had prevailed in PW (Xu et al., 2024; Yang et al., 2025; Yang et al., 2025). Genus-level shifts further illustrated this transition: the initially high abundances of *Pantoea*, *Pseudomonas*, and *Acinetobacter* in PW were drastically reduced in FPW. *Levilactobacillus*, *Lactobacillus*, *Enterococcus*, *Bacillus*, and *Weissella* became significantly enriched (Figure 3).

The observed shift in microbial community structure is critical for enhancing the quality and stability of the fermented feed. The substantial enrichment of LAB, particularly *Lactiplantibacillus* and Pediococcus, plays a dual role by reducing anti-nutritional factors and producing organic acids that suppress the growth of opportunistic pathogens and competitors present in PW, such as *Pantoea* and *Pseudomonas* (Xu et al., 2024; Yang et al., 2025; Yang et al., 2025). Therefore, the transition from a diverse, contaminant-heavy population in PW to an LAB-dominant community in FPW demonstrates that the fermentation process effectively inhibits undesirable microbes, thereby improving the preservation and nutritional profile of PW.

Notably, relative abundance derived from 16S rRNA sequencing cannot directly represent the actual metabolic activity of *Clostridium butyricum*. Lower relative abundance does not equal weaker butyrate-producing capacity. Although butyric acid is primarily produced by members of the genus *Clostridium*, FPW harbored a microbiota dominated by lactic acid bacteria rather than *Clostridium*. The higher butyrate concentration observed in FPW can be attributed to metabolic cross-feeding within the synthetic consortium. Lactic acid bacteria generate abundant lactate during fermentation, which acts as a critical precursor for butyrate synthesis by *Clostridium butyricum*. Even with moderate relative abundance of *C. butyricum*, sufficient lactate supply greatly promoted its butyrate-producing activity. In comparison, PW showed higher relative abundance of indigenous *C. butyricum* but lacked adequate lactate substrate, leading to lower butyrate accumulation. The lactic acid content was 1.43% in PW and 6.64% in FPW, respectively. It should be noted that acetic acid and propionic acid were not quantified in the present study, and the corresponding samples are no longer available for reanalysis, which represents a major limitation of this work. Determination of these key metabolites is required in future work to fully decipher the fermentation characteristics of pineapple waste.

**Figure 3:**
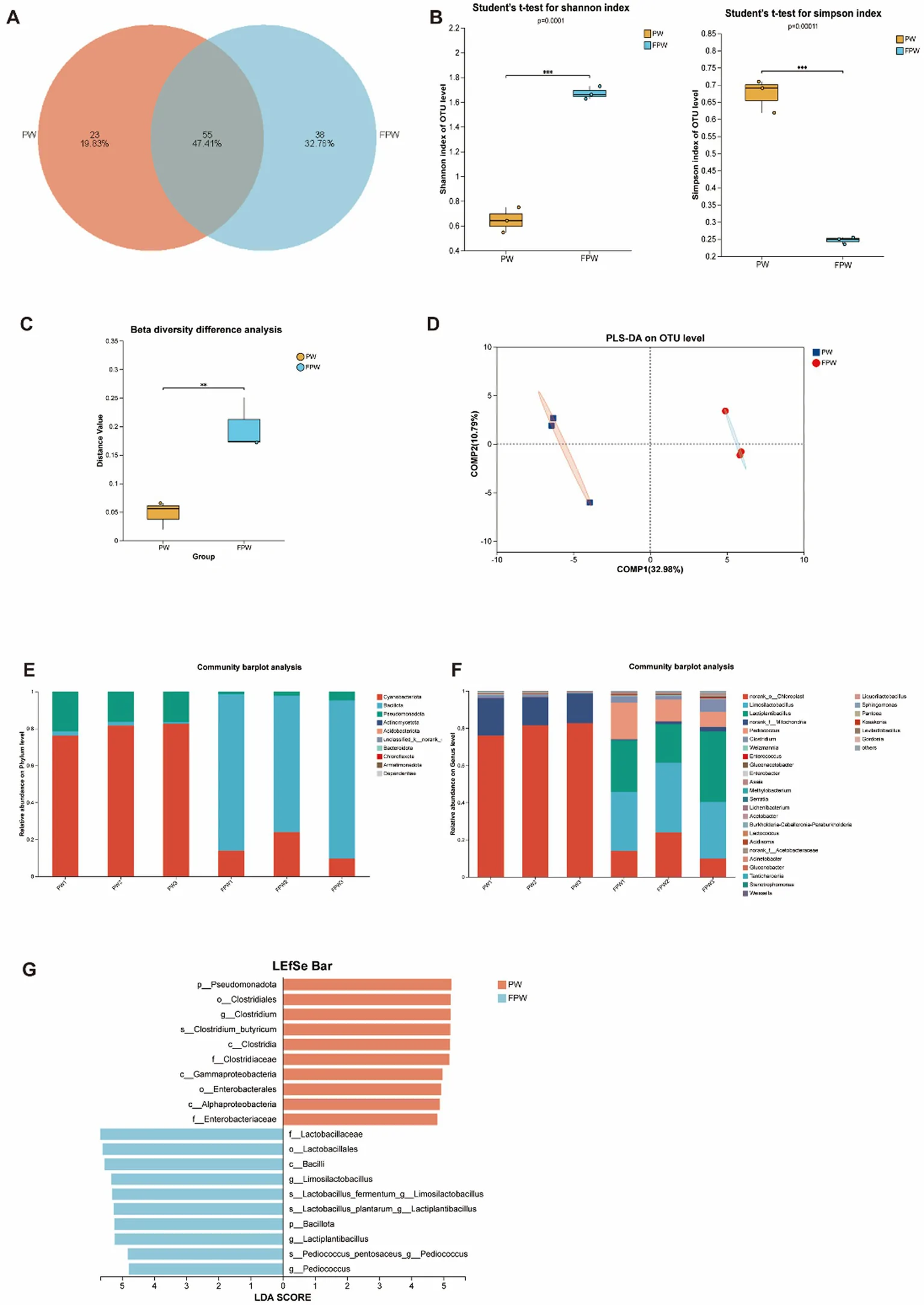
Microbial diversity and community analysis of fermented pineapple waste (FPW). **(A)** Venn diagram showing the overlap of OTUs between PW and FPW. **(B)** Student’s *t*-test for Shannon and Simpson indices at the OTU level. **(C)** Beta diversity difference analysis. **(D)** PLS-DA on OTU level. **(E)** Community barplot analysis at the Phylum level. **(F)** Community barplot analysis at the Genus level. **(G)** LEfSe bar showing LDA scores for differential taxa between PW and FPW. PW: Unfermented pineapple waste; FPW: Fermented pineapple waste.

### 3.4 Metabolite changes through fermentation

The metabolites before and after fermentation were clearly distinguishable (Figure 4). After fermentation, 258 metabolites were upregulated and 437 were downregulated. Most of the differential metabolites identified in this study were significantly upregulated following fermentation, including organic acids and their derivatives, lipids and lipid-like molecules, and organoheterocyclic compounds (Figures 4C–I). The primary metabolic pathways observed were closely related to amino acid metabolism (Figure 4D).

**Figure 4:**
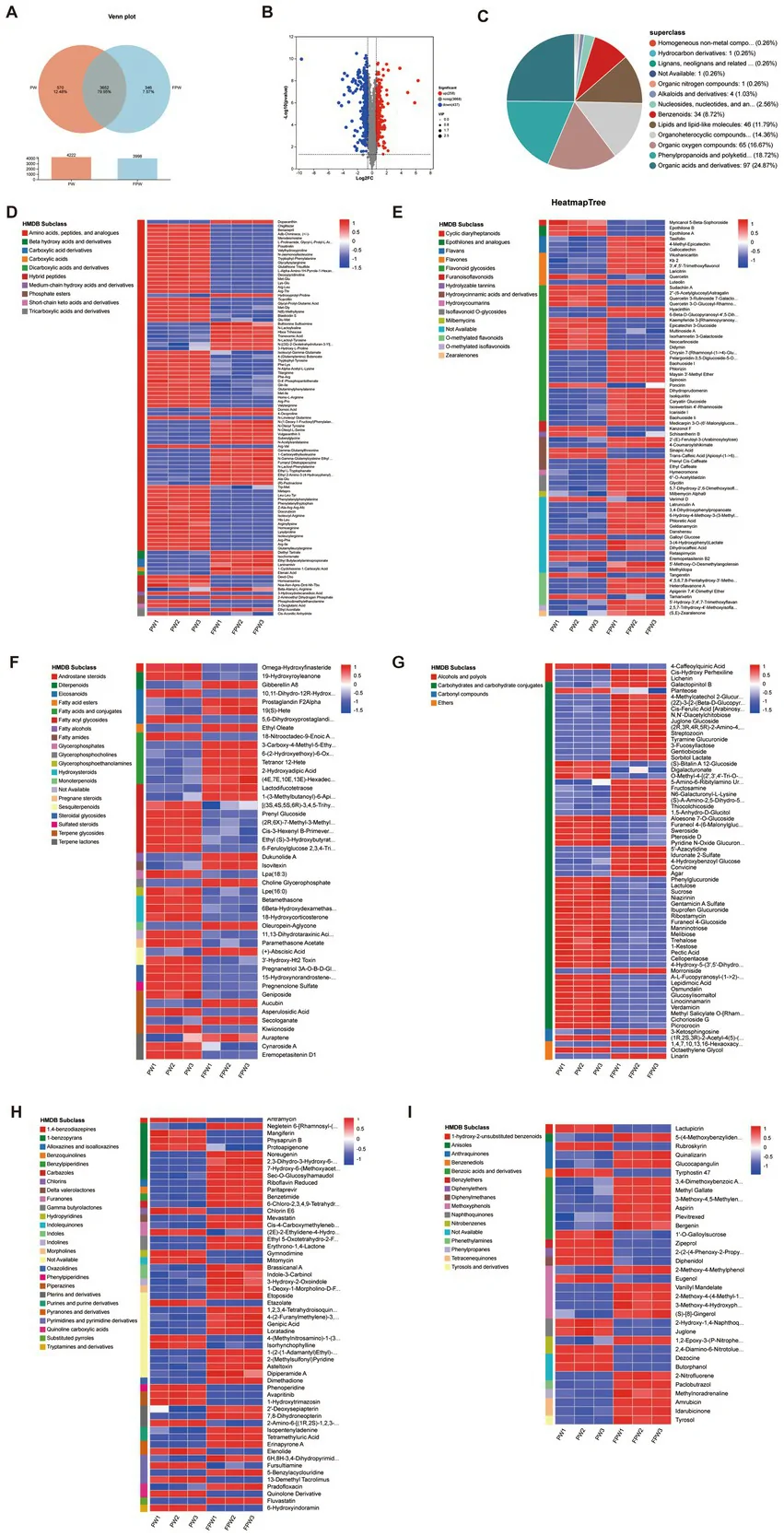
Metabolomic analysis of fermented pineapple waste (FPW) and its comparison with pineapple waste (PW). **(A)** Venn diagram showing the overlap of identified metabolites in PW and FPW. **(B)** Volcano plot of differential metabolites between PW and FPW. **(C)** Pie chart of metabolite classes based on HMDB subclass classification. **(D)** Heatmap of differential metabolites categorized as organic acids and derivatives. **(E)** Heatmap of differential metabolites categorized as phenylpropanoids and polyketides. **(F)** Heatmap of differential metabolites categorized as lipids and lipid-like molecules. **(G)** Heatmap of differential metabolites categorized as organic oxygen compounds. **(H)** Heatmap of differential metabolites categorized as organoheterocyclic compounds. **(I)** Heatmap of differential metabolites categorized as benzenoids. PW: unfermented pineapple waste; FPW: fermented pineapple waste.

LAB exhibits strong proteolytic activity. Decompose proteins into small peptides and free amino acids. Following fermentation, a wide array of essential amino acids (e.g., valine, phenylalanine, isoleucine) and non-essential amino acids (e.g., serine, Proline was significantly enriched; Figure 4D). These findings align with previous studies reporting amino acid enrichment during fermentation (Erem and Kilic-Akyilmaz, 2024; Yang et al., 2024). Specifically, *Bacillus* species, which are widely used in food and feed fermentation, demonstrate a robust capacity to convert agricultural by-products into rich sources of amino acids through the secretion of extracellular proteases (Liu et al., 2024; Mok et al., 2019). The enrichment of these compounds enhances both the nutritional value and the probiotic potential of the final product.

LAB metabolize carbohydrates to produce organic acids, which lower the substrate pH and suppress pathogenic microorganisms. In this study, glucose and fructose, the primary fermentable sugars, decreased markedly (Figure 4G), confirming their utilization by LAB. Conversely, the significant increase in succinic acid or related intermediates (Figure 4D) indicates enhanced microbial metabolic activity, as these are key metabolites of the tricarboxylic acid cycle (Bai et al., 2020; Li et al., 2023). Furthermore, notable reductions were observed in complex carbohydrates such as melezitose and α-lactose (Figure 4G). These changes are attributed to the secretion of extracellular enzymes such as cellulase and galactosidase by the fermentation consortia, which degrade complex carbohydrates into simpler sugars for microbial assimilation (Erem et al., 2024; Wang et al., 2021).

The fermentation process also significantly altered the lipid profile. Several Lactococcus and *Lactobacillus* species are known for high lipase production, which facilitates the hydrolysis of complex lipids into free fatty acids (Xia et al., 2023; Liu et al., 2024). This enzymatic activity explains the observed bidirectional changes in lipids and lipid-like molecules, where specific lipid species increased while others were depleted to provide carbon resources for microbial growth (Figure 4E).

The enrichment of bioactive compounds further adds to the functional value of the fermented product. In this study, metabolites involved in vitamin metabolism and secondary metabolite pathways showed significant enrichment (Figures 4H,I). This is often attributed to the microbial degradation of plant cell walls and the release of free phenolic acids via microbial esterases (Erem et al., 2024). These changes included the upregulation of specific organic acids and antioxidant-related derivatives (Figure 4F). Additionally, the enrichment of flavonoids such as luteolin, taxifolin, phlorizin, and gallocatechin highlights their physiological functions, Including antioxidant, inflammatory and immunomodulatory effects (Gu et al., 2025). These enhanced flavonoid levels play a role in promoting growth performance and supporting overall health in animal models fed fermented products.

### 3.5 Metabolomic profiling and functional correlation analysis of SysComs fermentation

To systematically elucidate the metabolic shifts and molecular mechanisms underlying the valorization of PW by the SysComs, untargeted metabolomic profiling was performed before and after fermentation (Figure 5). The SysComs fermentation dramatically reshaped the metabolome of PW, transforming raw agro-industrial residues into a nutrient-enriched, highly digestible fermented feed. KEGG pathway enrichment and topological analysis of the differential metabolites revealed profound alterations in carbohydrate, amino acid, and secondary metabolite pathways (Figure 5A). “Flavonoid biosynthesis” and “Flavone and flavonol biosynthesis” emerged as highly enriched pathways (*p* < 0.01) with exceptional topological impact scores (Impact Value > 0.35). This indicates a comprehensive biotransformation of polyphenolic matrices during fermentation. Quantitative assessment of key differential metabolites demonstrated a substantial enrichment of beneficial functional compounds in FPW compared to raw PW (Figure 5B). The relative abundances of key flavonoids and phenolics—including luteolin, taxifolin, phlorizin, and gallocatechin—increased drastically post-fermentation. This marked accumulation of secondary metabolites implies that the SysComs efficiently secreted glycoside hydrolases or esterases that cleaved complex plant cell-wall matrices or bound phenolics. This process liberated highly bioavailable flavonoids known for their potent antioxidant and anti-inflammatory properties in the piglet gut. Concurrently, significant variations were observed in organic acids, free amino acids, and small peptides (Figure 5C), reflecting the systemic degradation of anti-nutritional factors and macro-nutrients into easily absorbable nutrients. To uncover the functional division of labor and synergistic mechanisms within the SysComs, a Spearman correlation hierarchical clustering heatmap was constructed between the core bacterial genera and altered metabolites (Figure 5C). The structural members of the consortium displayed distinct, bifurcated correlation patterns. Key LAB, specifically *Limosilactobacillus* and *Lactiplantibacillus*, exhibited identical, highly synchronized profiles. They demonstrated extremely strong positive correlations (*p* < 0.01) with a dense cluster of beneficial metabolites, including hieracin, tyramine glucuronide, danshensu, and vital phenolic intermediates like luteolin and 2-Methoxy-4-(4-methyl-1,3-dioxolan-2-yl) phenol. Conversely, these LAB genera were strictly negatively correlated with structural sugars and certain anti-nutritional glycosides, such as sucrose, lactulose, and melibiose.

**Figure 5:**
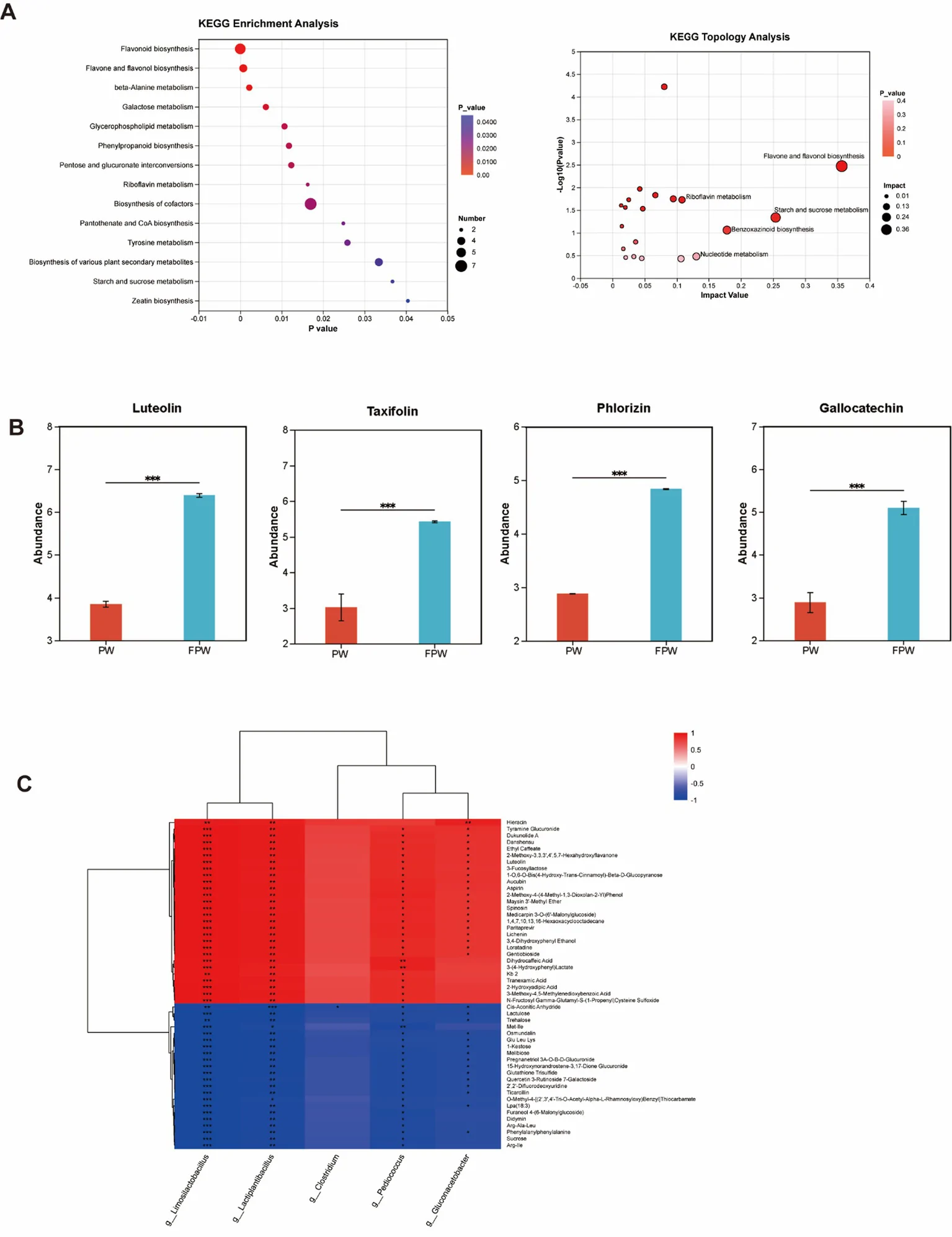
Metabolomics of fermented pineapple waste (FPW) before and after fermentation with SysComs. **(A)** KEGG pathway enrichment analysis and KEGG topological impact analysis of differential metabolites. **(B)** Relative abundances of key differential metabolites. **(C)** Heatmap of correlation analysis of differential metabolites and bacteria. PW: Pineapple waste, FPW: Fermented pineapple waste.

The untargeted metabolomic analysis revealed that SysComs fermentation profoundly reprogrammed the biochemical composition of PW, indicating a highly efficient microbial-driven valorization process. The significant enrichment of pathways related to flavonoid biosynthesis and flavone/flavonol metabolism suggests that fermentation not only improved nutrient accessibility but also enhanced the functional bioactivity of the substrate. Agricultural by-products such as PW typically contain substantial amounts of polyphenols in structurally bound or glycosylated forms, which limits their bioavailability in monogastric animals (Ruiz Rodríguez et al., 2021). The dramatic accumulation of free flavonoids, including luteolin, taxifolin, phlorizin, and gallocatechin, suggests that the microbial consortium effectively disrupted plant cell-wall complexes and hydrolyzed conjugated phenolic compounds, thereby releasing bioactive metabolites with enhanced physiological relevance (Gao et al., 2024; Muñoz et al., 2024).

These metabolite transformations likely contribute directly to the improved functional properties of FPW. Flavonoids such as luteolin and taxifolin are known to possess potent antioxidant, anti-inflammatory, and intestinal barrier-protective activities, which are particularly relevant for weaned piglets experiencing oxidative and inflammatory stress (Liang et al., 2024). The enrichment of these compounds may therefore provide a mechanistic explanation for the enhanced intestinal resilience and growth-promoting effects observed following FPW supplementation. In addition, the concurrent increase in organic acids, free amino acids, and small peptides indicates extensive microbial degradation of complex macromolecules into readily absorbable nutrients, further improving the nutritional quality of the fermented substrate (Luo et al., 2023). The microbe–metabolite correlation analysis further highlights the functional specialization and cooperative interactions within the SysComs. *Limosilactobacillus* and *Lactiplantibacillus* exhibited highly synchronized positive associations with numerous beneficial metabolites while showing negative correlations with residual sugars such as sucrose and melibiose, suggesting their central role in carbohydrate utilization, acidification, and phenolic biotransformation (Gao et al., 2024; Paventi et al., 2025). The acidic fermentation environment generated by these LAB likely promoted both enzymatic and non-enzymatic hydrolysis of plant-derived conjugates, accelerating nutrient release. This division of metabolic labor suggests that the consortium functioned as an integrated ecological unit rather than a simple additive combination of strains. The depletion of residual oligosaccharides and structural carbohydrates further supports efficient substrate depolymerization during fermentation. This reduction is nutritionally advantageous because poorly digestible carbohydrates and anti-nutritional factors in plant-derived feed ingredients can impair nutrient utilization and intestinal health in pigs. By converting these components into digestible nutrients and functional metabolites, SysComs fermentation substantially upgraded PW from a low-value agro-industrial residue into a biologically active feed ingredient. Collectively, these findings demonstrate that the beneficial effects of FPW arise from microbial metabolic remodeling. The synergistic interactions among consortium members enabled simultaneous nutrient liberation, anti-nutritional factor reduction, and generation of host-beneficial metabolites, thereby providing mechanistic support for the observed improvements in piglet performance and intestinal health.

### 3.6 Growth performance and digestibility

The efficacy of supplementing pig diets with FPW was assessed through growth performance across two production stages and apparent nutrient digestibility (Table 3). Throughout the experiment, pigs receiving FPW achieved a significantly higher ADG of 772.08 ± 12.23 g d^−1^ compared with 682.64 ± 31.02 g d^−1^ in the control (CON) group (*p* = 0.03), representing a 13% improvement. The PW group displayed intermediate ADG (708.65 ± 11.51 g d^−1^). The FCR was numerically lowest with FPW (2.02 ± 0.07) compared with CON group (2.20 ± 0.07). The ADFI remained statistically comparable across treatments, indicating that enhanced growth resulted from improved nutrient utilization rather than elevated feed consumption. The advantage of FPW was particularly evident in Stage-2, where ADG reached 807.57 ± 22.37 g d^−1^ (710.99 ± 26.99 g d^−1^ in CON; *p* = 0.03) with a trend toward improved FCR (2.10 ± 0.09 versus 2.28 ± 0.04; *p* = 0.09). Apparent digestibility of gross energy was significantly higher in the FPW group (87.46%) than in CON (85.36%), while crude protein digestibility showed a numerical but non-significant increase (84.76% versus 81.94%). These outcomes demonstrate that fermentation markedly enhances the nutritional value of pineapple waste.

**Table 3:** Effects of adding fermented pineapple waste to diet on pig growth performance and apparent digestibility (as-fed basis).

Items	CON	PW	FPW	*p* value
Stage-1				
Initial weight, kg	23.54 ± 0.49	23.39 ± 1.16	23.46 ± 0.95	0.99
Final weight, kg	33.86 ± 1.08	34.12 ± 1.24	35.07 ± 0.93	0.72
ADFI, g d^−1^	1,323.41 ± 19.80	1,370.67 ± 29.55	1,383.51 ± 40.31	0.11
ADG, g d^−1^	645.44 ± 39.34	670.59 ± 14.7	725.5 ± 1.39	0.39
FCR	2.08 ± 0.13	2.05 ± 0.08	1.91 ± 0.05	0.43
Stage-2				
Initial weight, kg	33.86 ± 1.08	34.12 ± 1.24	35.07 ± 0.93	0.72
Final weight, kg	48.79 ± 1.59	49.61 ± 1.43	52.03 ± 1.32	0.31
ADFI, g d^−1^	1,618.54 ± 33.69	1,734.40 ± 38.30	1,689.15 ± 38.09	0.14
ADG, g d^−1^	710.99 ± 26.99^b^	737.64 ± 10.28^b^	807.57 ± 22.37^a^	0.03
FCR	2.28 ± 0.04	2.35 ± 0.08	2.10 ± 0.09	0.09
All stage				
Initial weight, kg	23.54 ± 0.49	23.39 ± 1.16	23.46 ± 0.95	0.99
Final weight, kg	48.79 ± 1.59	49.61 ± 1.43	52.03 ± 1.32	0.31
ADFI, g d^−1^	1,493.86 ± 25.35	1,577.11 ± 30.99	1,560.22 ± 38.14	0.21
ADG, g d^−1^	682.64 ± 31.02^b^	708.65 ± 11.51^ab^	772.08 ± 12.23^a^	0.03
FCR	2.20 ± 0.07	2.23 ± 0.08	2.02 ± 0.07	0.17
Digestibility				
Gross energy, %	85.36 ± 0.33^b^	86.42 ± 0.67^ab^	87.46 ± 0.51^a^	0.04
Crude Protein, %	81.94 ± 1.45	83.10 ± 1.52	84.76 ± 1.45	0.39

Moisture content of diets in Stage-1: CON, 12.72%; PW, 12.79%; FPW, 12.80%. Moisture content of diets in Stage-2: CON, 12.52%; PW, 12.46%; FPW, 12.38%. In table, values within the same row bearing different superscript letters (a, b) are significantly different at *p* < 0.05, whereas values sharing the same letter are not significantly different.

The superior performance of FPW relative to both CON and PW underscore the mechanistic benefits of microbial fermentation. Solid-state or submerged fermentation likely hydrolyzes structural carbohydrates, liberates fermentable sugars, reduces anti-nutritional factors, and enriches the substrate with microbial enzymes (including residual bromelain activity) and probiotic metabolites. Consequently, energy and protein bioavailability increase, as reflected in the elevated gross-energy digestibility and improved feed efficiency. Gut microbiota modulation and short-chain fatty acid production may further contribute to enhanced intestinal health and nutrient absorption, consistent with observations in pigs supplemented with pineapple pomace (Yu et al., 2025).

These findings align with Deng et al. (2022), who reported improved ADG and economic returns in cattle fed fermented pineapple residue without compromising ruminal fermentation. In contrast, Vasconcelos et al. (2020) observed linear declines in ADG and ADFI when unfermented pineapple byproduct was included at high levels (27%) in finishing-pig diets, primarily due to elevated insoluble fiber. In addition, Liu et al. (2021) supplemented dairy cow diets with pineapple residue at low and high inclusion levels of 1.4 and 3.6 kg dry matter (DM)/day, respectively. The corresponding dry matter digestibility values were 73.8 and 63.8%, while total digestible nutrient (TDN) digestibility reached 70.3 and 63.4%. At high inclusion levels, nutrient digestibility was lower compared with low inclusion levels, though no significant overall reduction was observed. Both high- and low-dose feeding regimens achieved satisfactory feeding performance. This study found that supplementing the basal diet with 3.5% unfermented pineapple waste increased ADG and gross energy digestibility. Better performance was observed in the group fed the same inclusion level of fermented pineapple waste, which indicates that fermentation facilitates nutrient digestion and utilization in pigs. Broader reviews confirm that solid-state fermentation of agro-industrial wastes consistently improves palatability, digestibility, and animal performance while supporting circular-bioeconomy principles (Yafetto et al., 2023; He and Cui, 2025).

From a bioresource-technology perspective, FPW offers a low-cost, regionally available strategy to valorize the substantial pineapple waste stream generated in tropical production areas, simultaneously reducing environmental burdens associated with open dumping or uncontrolled decomposition and decreasing reliance on conventional protein and energy feeds. Potential limitations include the need to optimize inclusion rates for different growth phases and to evaluate long-term effects on carcass composition, meat quality, and gut microbiome stability. These results highlight the value of targeted microbial pretreatment for upgrading lignocellulosic fruit wastes into functional animal-feed ingredients and provide a foundation for subsequent economic and microbiome analyses.

### 3.7 Bacterial diversity of the gut microbiota of pig

Dietary supplementation with FPW significantly modulated the colonic microbial diversity and community structure in piglets compared with the CK and PW groups (Figure 6). Alpha diversity analysis, evaluated via the Kruskal-Wallis H test at the OTU level, revealed profound shifts in microbial richness (Figure 6A). Both the PW and FPW groups exhibited significantly higher Ace and Chao indices compared to the CK group. Notably, the median values of both indices reached their highest levels in the FPW group, indicating that the introduction of the SysComs via fermented feed successfully enriched and diversified the gut ecosystem without causing ecological dysbiosis. Beta diversity, assessed using partial least squares discriminant analysis (PLS-DA) at the OTU level, demonstrated distinct differences in community structures across treatments (Figure 6B). Samples from the CK, PW, and FPW groups clustered tightly within their respective groups and separated clearly along the first principal component (COMP1: 11.68%) and second principal component (COMP2: 6.43%). The marked spatial segregation of the FPW cluster from both the CK and PW groups indicates that the bioactive metabolites and prebiotic components present in FPW uniquely reshaped the gut microbial community. Community composition analysis at the phylum level showed that the gut microbiota was predominantly dominated by *Bacillota* (synonym *Firmicutes*) and *Bacteroidota*, which collectively accounted for over 90% of the total relative abundance across all groups (Figure 6C). At the genus level, the community barplot analysis highlighted a highly dynamic restructuring of core taxa (Figure 6D). The major identified genera included *Clostridium*, *Terrisporobacter*, *Muribaculaceae*, *Clostridia* UCG-014, and *Limosilactobacillus*. To identify specific biomarker taxa driving these community shifts, LEfSe analysis was performed (Figure 6E). The CK group was characterized by an enrichment of *Atopobiaceae*, *Olsenella*, and *Tractidigestivibacter*. The unfermented PW group was characterized by biomarkers such as RF39, and UCG-005. In contrast, the FPW group was selectively enriched with a robust cohort of signature biomarkers, including *Christensenellales*, *Christensenellaceae*, *Christensenellaceae* R-7 group, *Lachnospiraceae* NK4A136 group, and *Oxalobacter*, suggesting a selective proliferation of fiber-degraders and producers of beneficial SCFAs. The distinct shifts in key genera were further validated by a Kruskal-Wallis H test bar plot comparing mean proportions (Figure 6F). Significant differences were observed in several functional genera across the groups. Notably, the *Christensenellaceae* R-7 group exhibited a highly significant enrichment in the FPW group, with its mean proportion increasing to over 5% compared to less than 1.5% in the CK group (*p* < 0.01). Concurrently, the proportion of RF39 was prominently elevated in both the PW and FPW groups relative to the CK group (*p* < 0.05). Furthermore, the mean proportions of UCG-005, *Lachnospiraceae* NK4A136 group, *Tractidigestivibacter*, *Monoglobus*, *Olsenella*, *Oscillibacter*, *Adlercreutzia*, and *Senegalimassilia* all varied significantly across treatments (*p* < 0.05). Specifically, *Lachnospiraceae* NK4A136 group and *Monoglobus*—taxa renowned for their ability to hydrolyze complex plant carbohydrates and sustain intestinal mucosal integrity—maintained robust proportions in the FPW group. Taken together, these results demonstrate that SysComs-fermented pineapple waste works synergistically with the native microbiome. It establishes a highly diverse and favorable gut microbial architecture characterized by the enrichment of core functional homeostatic biomarkers.

**Figure 6:**
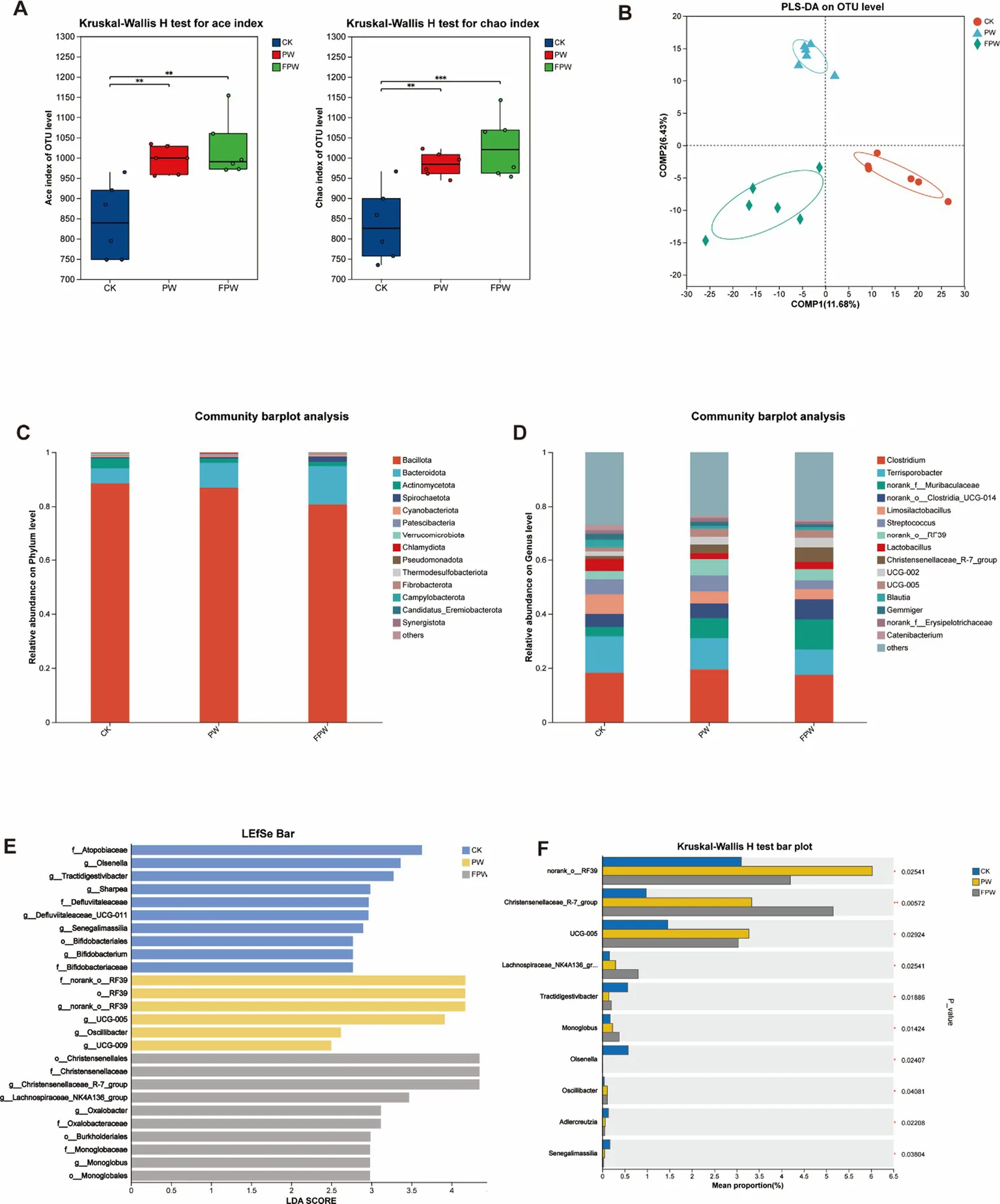
Microbial diversity analysis in animal trials with fermented pineapple waste (FPW). **(A)** Kruskal-Wallis H test for Ace and Chao indices at the OTU level. **(B)** PLS-DA analysis at the OTU level. **(C)** Community barplot analysis at the Phylum level. **(D)** Community barplot analysis at the Phylum level. **(D)** Community barplot analysis at the Genus level. **(E)** LEfSe bar showing LDA scores for differential taxa. **(F)** Kruskal-Wallis H test bar plot showing mean proportions of differential genera. CK: Control group; PW: Unfermented pineapple waste; FPW: Fermented pineapple waste.

The present findings demonstrate that dietary supplementation with FPW substantially remodeled the colonic microbial ecosystem of pigs, suggesting that the benefits of fermented pineapple waste extend beyond nutrient provision to active modulation of host gut microbiota (Zhang et al., 2023). The significant increase in alpha diversity, particularly the elevated Ace and Chao indices observed in the FPW group, indicates enhanced microbial richness and ecological complexity. Higher gut microbial diversity is generally considered a hallmark of intestinal resilience and metabolic stability, when pig often experience microbial disruption, intestinal inflammation, and reduced digestive efficiency (Gardiner et al., 2020; Liu et al., 2022). The fact that FPW further increased diversity compared with unfermented PW suggests that microbial fermentation substantially improved the ecological functionality of the substrate. The clear separation of microbial communities revealed by beta diversity analysis further supports the notion that FPW exerted a distinct microbiota-shaping effect rather than merely serving as a fiber supplement. This shift likely reflects the combined influence of fermentation-derived bioactive metabolites, partially degraded polysaccharides, organic acids, and residual beneficial microorganisms introduced through the synthetic microbial consortium. Such components may collectively create a more favorable intestinal niche that selectively supports beneficial microbial colonization while suppressing less adaptive taxa (Wang et al., 2021).

At the compositional level, the enrichment of *Christensenellaceae* R-7 group and *Lachnospiraceae* NK4A136 group in the FPW group is particularly noteworthy. Members of *Christensenellaceae* are increasingly recognized as beneficial commensals associated with gut metabolic health, intestinal homeostasis, and efficient fermentation of complex carbohydrates (Waters and Ley, 2019; Sebastià et al., 2024). Their expansion suggests enhanced fiber utilization and a healthier colonic fermentation environment. Similarly, *Lachnospiraceae* taxa are well-known producers of short-chain fatty acids, particularly butyrate, which serves as a critical energy substrate for colonocytes, strengthens epithelial barrier integrity, and exerts anti-inflammatory effects (Singh et al., 2023). The selective proliferation of these taxa strongly suggests that FPW promoted a microbial configuration favorable for intestinal health maintenance. The maintenance of *Monoglobus* abundance in the FPW group further reinforces this interpretation. *Monoglobus* species are specialized degraders of complex plant-derived polysaccharides, particularly pectin-rich substrates (Kim et al., 2019), which is highly relevant given the compositional characteristics of pineapple waste. Their persistence indicates that fermentation likely preserved or enhanced the accessibility of fermentable fiber fractions, thereby sustaining beneficial saccharolytic fermentation in the hindgut. This is particularly advantageous for weaned piglets, whose immature digestive systems often struggle to efficiently utilize structurally complex plant materials.

Conversely, the enrichment of *Olsenella* and *Tractidigestivibacter* in the control group may reflect a less favorable microbial state. Although some members of these genera participate in carbohydrate metabolism, their increased abundance has also been associated in certain contexts with microbial imbalance or altered fermentation profiles under nutritional stress. The displacement of these taxa in the FPW group suggests that fermented supplementation shifted the microbial ecosystem toward a more functionally beneficial and metabolically balanced structure. Interestingly, unfermented PW also altered microbial composition, particularly through the enrichment of RF39-related taxa, indicating that pineapple waste itself possesses prebiotic potential due to its fiber and residual phytochemical content. However, the more pronounced enrichment of SCFA-associated and fiber-degrading taxa in the FPW group indicates that microbial fermentation substantially amplified this effect. This suggests that the SysComs-mediated biotransformation enhanced substrate digestibility and generated a more selective ecological pressure favoring beneficial colonizers.

Collectively, these findings suggest that FPW acts as a microbiota-targeted functional feed ingredient capable of improving intestinal microbial diversity, promoting beneficial carbohydrate-fermenting and SCFA-producing taxa, and supporting gut ecological stability during weaning stress. These microbiota shifts likely represent an important mechanistic basis for the observed improvements in piglet intestinal health and growth performance. The modulation of gut microbiota related to lactate metabolism observed with thermophile-fermented feed in pigs further supports the potential of fermented substrates to reshape microbial metabolic pathways in the hindgut (Yoshikawa et al., 2024).

## 4 Conclusion

In conclusion, this study demonstrated that SysComs-fermented pineapple waste (FPW) improved nutritional profiles, reshaped microbial community structure, and increased the diversity and abundance of beneficial metabolites. Dietary supplementation with FPW did not impair the growth performance of pigs and exerted superior beneficial effects compared with untreated raw pineapple waste. These findings provide a strong theoretical foundation for developing feed products derived from FPW and expand the pool of available alternative feed ingredients. Meanwhile, the present study has several limitations, including a single inclusion level of fermented pineapple waste, a relatively short feeding trial period, and the absence of quantitative assays and *in vitro* functional verification. Future research will focus on scaling up fermentation processes, conducting long-term feeding trials, and assess environmental impacts, with an emphasis on the circular economy and to further promote low-carbon recycling of agricultural waste.

## Data Availability

The sequencing data generated in this study have been deposited in the NCBI Sequence Read Archive (SRA) under accession number “PRJNA1527545”.

## Footnotes

1 https://v.majorbio.com/project-center/overview.
